# Percutaneous radiofrequency ablation of adrenal metastases from hepatocellular carcinoma: a single-center experience

**DOI:** 10.1186/s40644-019-0231-7

**Published:** 2019-06-26

**Authors:** Jingzhi Huang, Xiaohua Xie, Jinhua Lin, Wei Wang, Xiaoer Zhang, Ming Liu, Xiaoju Li, Guangliang Huang, Baoxian Liu, Xiaoyan Xie

**Affiliations:** Department of Medical Ultrasound, Division of Interventional Ultrasound, The First Affiliated Hospital, Sun Yat-sen University, No. 58 Zhongshan 2nd Road, Guangzhou, 510080 China

**Keywords:** Adrenal metastasis, Hepatocellular carcinoma, Ultrasound, Radiofrequency ablation

## Abstract

**Background:**

The prognosis of adrenal metastases (AM) from hepatocellular carcinoma (HCC) with surgical contraindication was poor. This study evaluated the feasibility, safety and treatment efficacy of percutaneous ultrasound (US)-guided radiofrequency ablation (RFA) for the local treatment of AM originated from HCC.

**Methods:**

A retrospective study was carried out on 22 patients (21 male and 1 female, mean age, 53.0 ± 13.0 years) who had single AM (mean diameter, 4.0 ± 1.8 cm, range, 1.7–8.0 cm) originated from HCC and received US-guided percutaneous RFA at our institution. The diagnosis was established on typical radiologic findings. The primary technical success was defined as the tumour being completely ablated in the first RFA session. The secondary technical success was defined as tumour residual left from the first ablation was completely ablated by a second ablation session. Local tumour progression (LTP) and overall survival (OS) were estimated by using Kaplan-Meier analysis.

**Results:**

A total of 25 ablation sessions were performed. The primary technical success and the secondary technical success were 77.3% (17 of 22) and 86.4% (19 of 22), respectively, with the major complication rate at 4.5% (1 of 22). The median follow-up period after RFA was 10 months (3–55 months). During the follow-up period, five patients were detected LTP. The LTP at 3, 6, and 12 months were 15.8, 26.3, and 26.3%, respectively. Nine patients died of distant extra-adrenal metastases and another five of liver failure due to HCC. The OS at 6, 12, 24 months after RFA for AM were at 79.7, 52.6, and 32.9%, respectively.

**Conclusion:**

Percutaneous US-guided RFA in the treatment of AM originated from HCC is feasible, safe and effective.

## Introduction

Hepatocellular carcinoma (HCC) ranks the second by absolute years of life lost as population aging and growth around the world [1]. Meanwhile, with the development of medical technology, the early diagnosis and radical treatment of HCC made an increase of survival. However, extrahepatic metastases become common in the long term follow-up. One of the common metastatic sites is adrenal gland via hematogenous metastasis; especially more commonly seen at the right adrenal gland [2–4]. The prognosis of HCC adrenal metastases without effective treatment is poor, with the OS at around 5.64 months [5, 6]. Many studies have reported that the patients suffered HCC with adrenal metastasis (AM) may benefit from adrenalectomy [7–9]. Nevertheless, few patients are eligible for surgical treatment, due to previous hepatectomy or existence of extra-adrenal tumours. Previous studies have reported that transhepatic arterial chemembolization (TACE) is a feasible and safe alternative in the management of HCC with adrenal metastasis. But the TACE procedure is technically difficult since the feeding arteries are too thin to be superselective, as the feeding vessels consisted of three vessels: inferior phrenic artery, aorta and renal artery. Percutaneous ethanol injection therapy (PEIT) is also used for treating small lesions in adrenal with satisfactory outcomes. But for large lesions, ethanol is difficult to diffuse fully within the tumour. Besides, for malignant lesions, higher incidence of residual tumour and recurrence are common [2, 6, 10].

Recently, image-guided ablation therapy such as radiofrequency ablation (RFA), microwave ablation (MWA) and cryoablation has shown promising results in both primary and metastatic adrenal neoplasms [11–13]. Previous reports have shown that RFA on functioning adrenal adenomas may achieve normalization of hormone secretion [14].

In addition, many studies have reported that RFA may achieve 77–80% of local tumour control in AMs originated from lung or kidney [15–18]. However, there are few studies focus on RFA for the AMs originated from HCC.

Therefore, the purpose of this study was to summarize our single-center experience of percutaneous ultrasound (US)-guided RFA for the local treatment of AM originated from HCC and to evaluate its feasibility, safety and treatment efficacy.

## Methods

### Patients

This retrospective study was approved by the Ethics Committee of our hospital, which was conformed to the standards of the Declaration of Helsinki. Informed consent had been obtained from all patients before the ablation procedure. Informed consent for participation in this retrospective study was waived by the institutional review board.

From September 2008 to September 2018, US-guided percutaneous RFA was performed on 22 patients with single AM originated from HCC at our institution. Patients were referred for ablation by a multi-disciplinary team, because of abdominal adhesion due to repeated surgical operations (*n* = 5), poor hepatic reserve (*n* = 9), existence of extra-hepatic and adrenal metastasis (*n* = 2), failure of radiotherapy for AM (*n* = 2), and refusal of surgical operation (*n* = 4). The inclusion criteria were as follows: (1) aged 18–75 years; (2) confirmed diagnosis of AM, with the maximum diameter less than 10 cm [17]; 3) for curative intent, defined as patients with single AM without intrahepatic recurrence or with limited intrahepatic recurrence which could be ablated simultaneously; for palliative intent, defined as patients with unilateral AM received debulking treatment for symptom relief in view of stable extrahepatic metastases; (4) an Eastern Cooperative Oncology Group (ECOG) performance status score of 0 or 1. Exclusion criteria were: (1) coagulation dysfunction with prothrombin time less than 25 s and platelet count below 50 × 10^9^ cells/L; (2) uncontrollable local or systemic infection; (3) contraindication for RFA due to cardiopulmonary dysfunction.

Synchronous metastasis was defined as AM detected ≤6 months after treatment of the primary tumour. Metachronous metastasis was defined as AM detected > 6 months after treatment of the primary tumour [19]. In all cases, patients were diagnosed as AM originated from HCC by clinical criteria. That was, HCC history with adrenal new lesion noted by at least two types of enhanced imaging, including contrast-enhanced computed tomography (CECT), dynamic contrast-enhanced magnetic resonance imaging (DCE-MRI) or contrast-enhanced ultrasound (CEUS) which had typical radiologic findings and increase in size on serial images [20, 21]. Apart from image examination, patients who were suspected of adrenal gland hyperplasia were also ruled out by means of serum hormone levels before the procedure.

### Pre-operative preparation

All the patients were hospitalized to have the blood pressure and heart rate stabilized prior to the RFA treatment. Comprehensive preoperative examinations such as blood count, biochemistry analysis, coagulation function, electrocardiograph (ECG), and X-ray were carried out to exclude ablation contraindications. Conventional US and CEUS were performed on each individual to determine the target tumour location, size, blood supply, and peripheral status of the lesion.

### RFA procedures

Percutaneous RFA was guided and monitored by real-time US using Acuson Sequoia 512 (Siemens Medical Solutions, Mountain View, CA, USA) equipped with a 4 V1 vector transducer (frequency range, 1.0–4.0 MHz), or Aplio 500 (Toshiba Medical Systems, Tokyo, Japan) equipped with a PVT-375 BT convex transducer (frequency range, 1.9–6.0 MHz). RFA was performed using a Cool-tip™ RFA system (Valleylab, Boulder, Colo, USA), which was consisted of a RF generator with a maximum power of 200 watts, a 17-gauge internally cooled electrode, and 2 dispersive pads. Two interventional radiologists (X.Y.X. and X.H.X., both with more than 20 years’ experience of tumour ablation) performed all the RFA procedures.

For AM located on the left side, the patient was placed in the right decubitus position, and a posterior path was recommended to avoid injury of the pancreatic cauda, spleen, or gastric fundus; for right-sided AM, the patient was placed in the left decubitus position or in the supine position, and a trans-hepatic approach was chosen. Local anaesthesia with 1% lidocaine in combination with conscious analgesic sedation by intravenous administration of fentanyl citrate and droperidol was used during the procedure. Under the real-time US guidance, the electrodes were introduced into the tumours. For tumours smaller than 3.0 cm in diameter, ablation with one insertion into the central portion was used; for tumours greater than 3.0 cm in diameter, overlapping ablations with two to three insertions were performed with inter electrode distance of 2.5–3.0 cm. The deepest portion of the tumour was first ablated, then the electrode was withdrawn about 1.5–2.0 cm to ablate the more superficial portion of the tumour. The ablation region was carefully monitored by real-time US, and complete treatment was considered if the hyperechoic region covered the entire lesion. When the target tumour abutted important structures, additional small doses of ethanol were injected via an 18-gauge needle to displace such adjacent structures before RFA. After ablation was completed, the puncture track was carefully ablation with the electrode being retracted by 1 cm increments to prevent bleeding and tumour seeding.

### Intra-operative monitoring

Intravenous access was established, BP was measured non-invasively and oxygen was administered via a nasal catheter. During the procedure, the patient’s BP, heart rate, ECG, and peripheral blood oxygen saturation level were carefully monitored. Emergency drugs such as nitroglycerin, nifedipine, metoprolol, and atropine were available for immediate administration.

### Post-operative management

After ablation, patients were carefully observed for 30 min and then returned to the ward if they were in stable condition. Vital signs were closely monitored for the first 6 h after having been returned to the ward. Clinical symptoms and complications were closely observed during the post-RFA hospital stay. Complications were reported using the standardized Society of Interventional Radiology (SIR) grading system. SIR-A and SIR-B were defined as mild complications; and SIR C-F was defined as major complications [22].

### Outcome assessment and follow-up

The primary technical success was defined as the tumour being treated according to protocol and disappearance of tumour enhancement on the initial contrast-enhanced images obtained 1 month after RFA [17]. In the case of viable residual tumour, additional ablation was given aiming for complete ablation (CA). The secondary success was defined as technical success determined on contrast enhanced images obtained 1 month after the second adrenal RFA for residual tumour. Technical feasibility was defined as imaging confirmation of complete tumour ablation with no visible residual tumour either after the first ablation or after a second ablation [17]. If the residual tumour was still viable after the additional ablation, RFA for AM was considered a failure and the patient was referred for other therapies.

The patients with CA were followed up at 3, 6, and 12 months for the first year, and 6 monthly thereafter or at any time if necessary. Local tumour progression (LTP) was defined as reappearance of enhancing tumour foci adjacent to the ablated zone on the contrast-enhance imaging findings during follow-up after the confirmation of CA. Overall survival (OS) was defined as the time from the first session of RFA for AM to death or censoring. Follow-up ended with the most recent clinic visit or the most recent imaging before September 2018 or at the death of patient.

### Statistical analysis

Continuous variables were presented as means ± standard deviation and compared using *t* test or Mann-Whitney test. Categorical variables were presented as numbers and percentages and compared using χ^2^ test or Fisher’s exact test. LTP rates and survivals were calculated using the Kaplan-Meier method. A two-tailed *P* value less than 0.05 was considered to indicate a statistically significant difference. The above statistical analysis was performed with SPSS 23.0 (SPSS Inc., Chicago, IL, USA).

## Results

### Patient and treatment characteristics

In total, 22 patients (21 male and 1 female) with 22 AMs originated from HCC were included in the present study. The mean age was 53.0 ± 13.0 years (range, 33–75 years). 20 patients were with a single metastatic tumour in the right adrenal gland and 2 in the left. The mean diameter of AM was 4.0 ± 1.8 cm (range, 1.7–8.0 cm). Seventeen tumours were ≤ 5 cm and 5 tumours were > 5 cm. Of the 20 AM patients who were treated with curative intent, 11 had isolated AM and 9 had concomitant limited intrahepatic tumours which could be ablated simultaneously. The remaining 2 patients with HCC metastases in adrenal gland and other sites (one had lung metastases and the other had bone and right iliac fossa metastases) were treated with palliative intent. Some of the patients received additional treatment: 3 patients had systemic therapy for AM before RFA, including chemotherapy (*n* = 1), cell immunetherapy (*n* = 1) and targeted therapy (*n* = 1); 9 patients with AM abutted important structures were administrated with additional ethanol injection and 1 patient having an AM at 6.4 cm in diameter was administrated with additional TACE before RFA. Clinical characteristics of these included patients are summarized in Table 1.

**Table 1:** Patient and adrenal metastasis characteristics

Characteristics	Value (*n* = 22)
Patient characteristics	
Sex	
Male	21
Female	1
Age, year^a^	52 (33–75)
Hepatitis	
Yes	19
No	3
Previous HCC treatment	
Resection	17
Transplantation	3
Ablation	2
AFP (μg/L)	
≤ 400	15
> 400	7
Cirrhosis	
Yes	10
No	12
Child-Pugh class	
A	19
B	3
ECOG Performance Status	
0	20
1	2
Tumour characteristics	
Time to adrenal metastases	
Synchronous (≤ 6 months)	6
Metachronous (> 6 months)	16
Extent of disease at time of ablation	
Isolated AM	11
rHCC and AM	9
Metastases in other sites	2
Tumour size	
≤ 5 cm	17
> 5 cm	5
Laterality	
Right	20
Left	2

^a^median (range), *HCC* hepatocellular carcinoma, *AFP* alpha-fetoprotein, *ECOG* Eastern Cooperative Oncology Group, *AM* adrenal metastasis, *rHCC* recurrent hepatocellular carcinoma

### Side effects and complications

There were three (3/22, 9%) patients who showed systolic BP over 180 mmHg during the ablation procedure were diagnosed as hypertension crisis. All of them were gave sublingual nitroglycerin to lower the BP. After the symptomatic treatments, the BP of 2 patients was normalized subsequently and the procedure continued with stable vital sign, while the remaining one patient without improvement on BP had the second session on following day. Two patients had heart rate at 37–39 beats per minute (bpm). After the intravenous administration of atropine, one patient was restored to a normal heart rate and the ablation procedure continued. The other patient was detected heart rate soaring to 145 bpm with frequent ventricular fibrillation. It caused an immediate termination of ablation. Moreover, a transient increase of Cardiac Troponin I was observed on this patient after returned to the ward. This patient is the only one who had major complication (SIR C) as myocardial transient ischemia. After given myocardial nutrient therapy 1 month later, the patient had the second ablation session with stable heart rate and achieved CA. No other ablation related complications such as intraoperative bleeding or thermal injury were observed in this study. Two patients complained a post-operative local pain, and were relieved by painkiller.

### Clinical outcomes

**Fig. 1:**
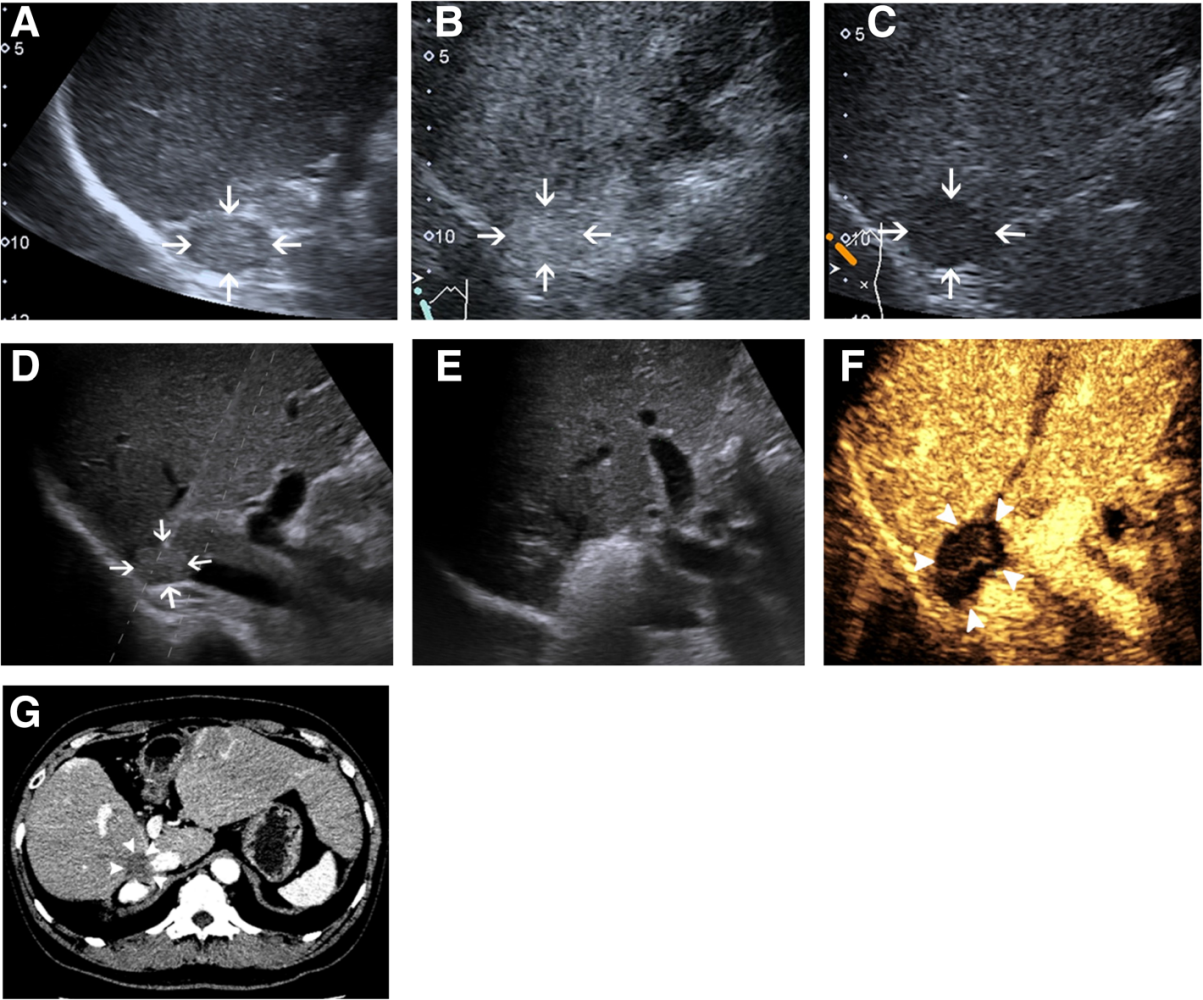
Images in a 46-year-old male patient received radiofrequency ablation (RFA) for adrenal metastasis (AM) originated from hepatocellular carcinoma (HCC). **a** Pre-interventional B-mode ultrasound showed a round mass at the upper pole of the right kidney (arrow). **b** and **c** contrast-enhanced ultrasound (CEUS) before RFA showed obvious enhancement in the early phase (**b**) and washout in the late phase (**c**). **d** Prober placement during the ablation of the right adrenal metastasis. **e** Tumour appeared completely hyperechoic after the beginning of RFA. **f** CEUS obtained 1 month after RFA showed no contrast enhancement, suggesting complete ablation. **g** Contrast-enhanced CT obtained 1 month after RFA showed no contrast enhancement, suggesting complete ablation

**Fig. 2:**
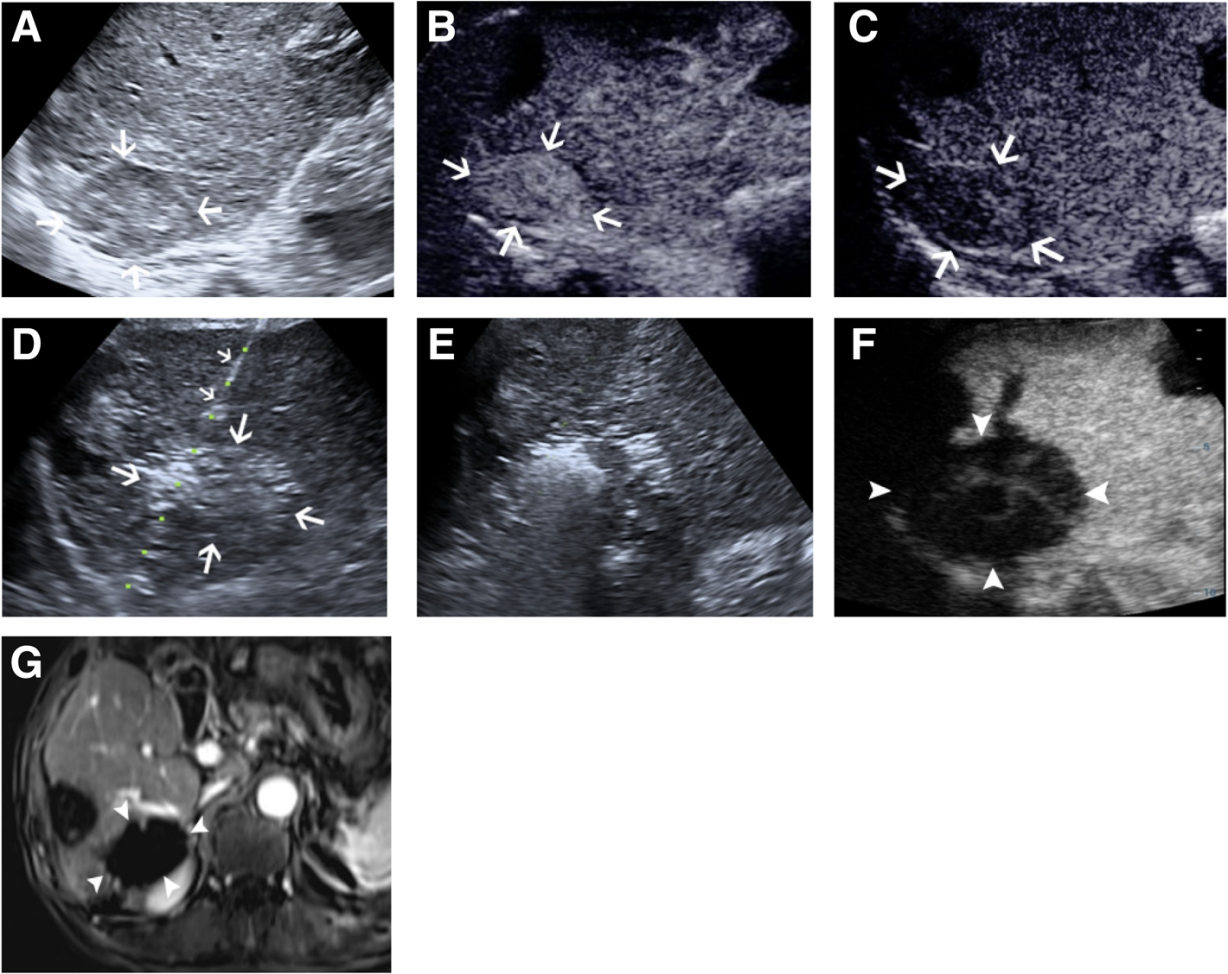
Radiofrequency ablation (RFA) in a 66-year-old man with metastatic hepatocellular carcinoma in the right adrenal gland. **a** Pre-interventional B-mode ultrasound showed a round mass at the upper pole of the right kidney (arrow). **b** and **c** contrast-enhanced ultrasound (CEUS) before RFA showed obvious enhancement in the early phase (**b**) and washout in the late phase (**c**). **d** Prober placement during the ablation of the right adrenal metastasis. **e** Tumour appeared completely hyperechoic after the beginning of RFA. **f** Follow-up CEUS 1 month after RFA showed no contrast enhancement, suggesting complete ablation. **g** Follow-up MRI 1 month after RFA showed no enhancement of the target tumour

A total of 25 sessions of RFA were performed on 22 patients with AM originated from HCC. Two patients had the ablation procedure suspended due to severe intraoperative fluctuations of BP and heart rate. Three patients had incomplete ablation with AM at 6.8–8.0 cm in diameter. The remaining 17 patients had completed ablated in the first session of RFA (Figs. 1 and 2), and the primary technical success was 77.3% (17 of 22). Tumour enhancement disappeared after the second session of RFA on both of the patients who suffered intraoperative adverse reactions. Among the 3 patients with residual lesions, only one patient received an additional RFA, but the tumour enhancement remained on CEUS at the tumour periphery. Therefore, the second technical success rate was 86.4% (19 of 22).

**Fig. 3:**
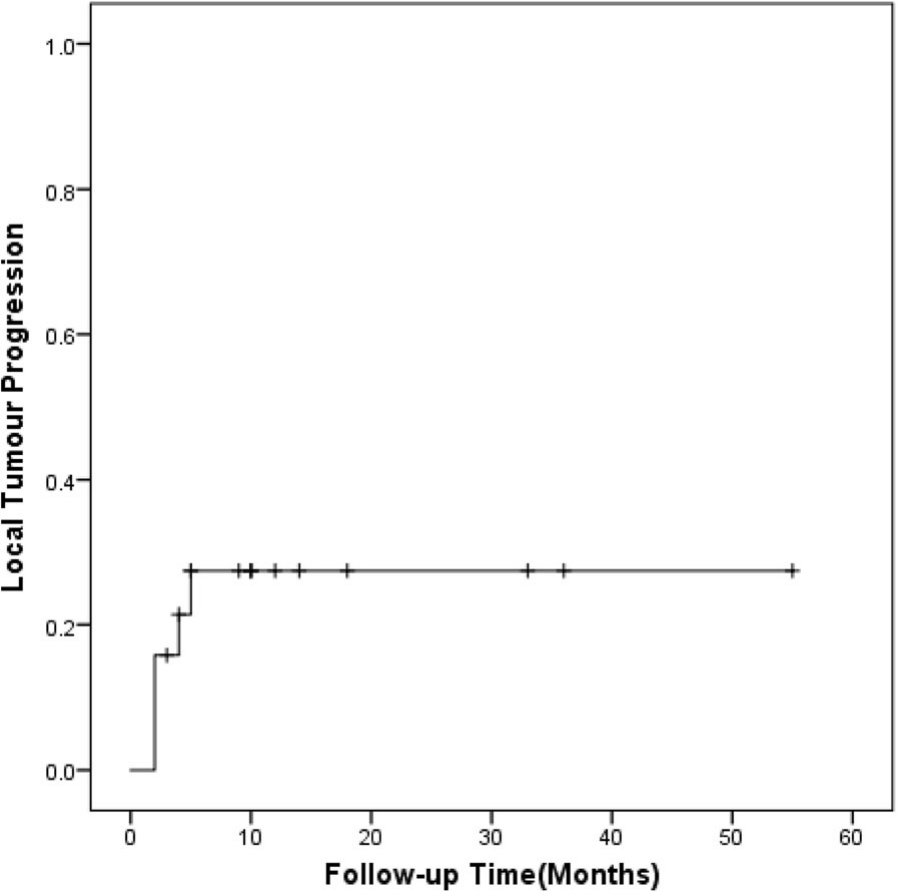
Local progression-free survival in successfully ablated AM tumors (*n* = 19)

Finally, a total of 19 patients entered the follow-up. The median follow-up period after RFA was 10 months (range, 3–55 months). During the follow-up period, five of 19 patients (26.3%) experienced LTP after RFA. The LTP rate at 3, 6, and 12 months were 15.8, 26.3, and 26.3%, respectively (Fig. 3). But none of them had additional treatment for LTP due to poor body condition.

**Fig. 4:**
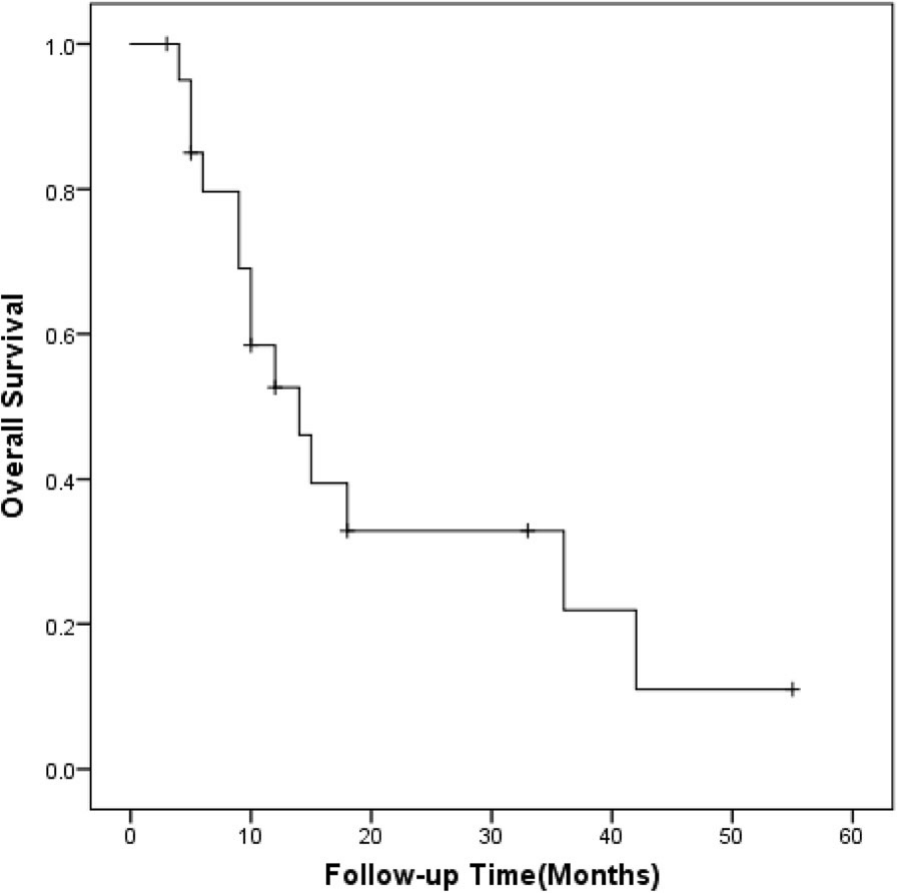
Overall survival after US-guided RFA of AM form HCC (*n* = 22)

At completion of the follow-up (September 2018), nine patients died of distant disease progression and five patents died of liver failure. One patient was lost after 5 months of follow-up. The median OS time was 14 months (95% confidence interval [CI], 7.9–20.1 months). The OS rate at 6, 12, 24 months after RFA for AM were 79.7, 52.6, and 32.9%, respectively (Fig. 4).

## Discussion

In the present study, we summarized our experience of percutaneous US-guided RFA in local treatment of AM originated from HCC and evaluated the feasibility, safety and treatment efficacy of such therapeutic method. Results of our study demonstrated that the procedure was relatively safe, with only one patient suffered major complication (SIR C). The technical success among patients with AM ≤5 cm had achieved 100% complete ablation. Among the patients with complete ablation, five patients experienced LTP, with the acceptable LTP rate of 26.3%. Therefore, we consider that US-guided RFA is a feasible and safe therapeutic option for AM originated from HCC.

As reported in previous studies, patients with AM treated by RFA showed local tumour control rate at 77.2–94% and the median survival time at 14.0–26.0 months, depending on origins of primary tumour [15–18, 23]. In addition, RFA may provide favourable treatment efficacy for ‘oligometastatic’ tumours from HCC. To date, few studies focused on evaluating the treatment outcomes of thermal ablation for AM from HCC. Kuehl et al. [24] demonstrated the CT-guided transhepatic RFA of right adrenal HCC metastases but with only 2 cases. It was considered that CT-guided RFA might be a feasible treatment in patients unable to undergo surgical resection. Recently, in another study by Yuan et al. [25], the treatment for AM from HCC was compared with a combined TACE-RFA and TACE alone. According to Yuan et al., RFA was not considered as a primary but an additional technique in the treatment of AM. The treatment efficacy of RFA alone was unclear in above study. In our opinion, it is necessary to conclude the experience and treatment outcomes of RFA for AM originated from HCC.

Previous studies showed that RFA was able to achieve technique successful rate between 77.2 and 100% in the adrenal metastasis and functioning adrenal adenomas [14–18]. In our study, the technical success was 86.4% (19 of 22). The cases failed in the completed ablation in our study were with AM > 5 cm. It hints that for AM > 5 cm, it is hard for RFA to achieve complete ablation. For tumours > 5 cm, combination treatment strategies should be considered. Several studies have reported that completed ablation of AM at 3.5–8.0 cm in diameter can be achieved with combined RFA and TACE [25, 26]. As reported, TACE blocks blood flow, hence lowered the cooling effect of circulation during RFA and reduced the loss of heat [25].

In the present study, the local tumour progression rate was 26.3%, which was similar to the previous studies of thermal ablation for AM [17]. Moreover, RFA is easy to repeat with low complications to achieve complete necrosis of AM, as long as the patient could tolerate. Surgery for resectable AM has been reported to have local tumour control rate of 77–83% [27–29]. However, in our knowledge, there are no studies directly compared these two treatment strategies for AM, due to the different inclusion criteria and heterogeneous baseline information. Perhaps, prospective, randomized, and multi-center controlled studies with appropriate sample size are helpful in the comparison of RFA and other treatments such as adrenalectomy.

Compared with CT/MRI, US is non-radiative, portable and real-time for guiding and monitoring during the interventional procedure. The right adrenal gland located between the liver and right kidney is less likely shaded from intestinal tract and bowl gas. Therefore, using real-time ultrasound guidance provides accurate imaging of the location of electrode inside the lesion and avoids damage of neighbouring tissue. As for left adrenal gland, which adjoins to intestinal canal and spleen, it is more likely to have piercing damage and thermal injury during ablation. In our study, ethanol was injected to displace abutted important structures. Besides, previous studies also showed that artificial ascites can be applied to separate adjacent structures from the tumour and the ablation zone without deputy injury [30]. In our study, no patient suffered electrode puncture related injury and thermal injury of adjacent tissues.

Hypertension crisis has been reported to be the main adverse reaction during adrenal ablation with incidence rate of 5.6–46% [11, 17], due to the different hypertension crisis criteria. Our study showed that three patients suffered from souring BP over 180 mmHg and the administration of vasodilators quickly relieved the symptom. Adrenal gland as one of significant endocrine organs in human body can release catecholamine to regulate blood pressure. Thus, it is important to monitor the BP and timely administer the adrenergic blocking medicines during the adrenal ablation. Two patients suffered heart rate decreasing < 40 bpm. Nevertheless, after injecting the same amount atropine, one patient was restored to normal heart rate while the other suffered frequent ventricular fibrillation. Perhaps, the medicine should be given more individually and the surveillance cannot be ignored.

Overall, our study had several limitations. Firstly, it was retrospective in design with a small sample size. Patients previously had different HCC treatment and various episodes of intrahepatic recurrence might result in bias of clinical outcomes. Secondly, patients were lack of pathologic examination before AM ablation in our study. But all the patients had clinical diagnosis confirmation with typical image before procedure. Thirdly, some patients had short follow-up period to explore AM recurrence. Finally, there was no matched control group of patients treated by surgery for comparison, which could further validate the clinical application of RFA as a treatment option for AM.

## Conclusions

In conclusion, our results demonstrated that RFA is feasible, safe and effective in the treatment of AM originated from HCC.

## Data Availability

Data sharing not applicable to this article as no datasets were generated.

## Abbreviations

AM: Adrenal metastases
BP: Blood pressure
CEUS: Contrast-enhanced ultrasound
CT: Computed tomography
ECG: Electrocardiograph
HCC: Hepatocellular carcinoma
LTP: Local tumour progression
MRI: Magnetic resonance imaging
MWA: Microwave ablation
OS: Overall survival
PEIT: Percutaneous ethanol injection therapy
RFA: Radiofrequency ablation
TACE: Transhepatic arterial chemembolization
US: Ultrasound
